# Circular RNA circ‐TNRC6B inhibits the proliferation and invasion of esophageal squamous cell carcinoma cells by regulating the miR‐452‐5p/DAG1 axis

**DOI:** 10.1002/1878-0261.13432

**Published:** 2023-04-16

**Authors:** Ruiyu Xu, Pingan Ding, Xiaoru Zhao, Ziyi Li, Fei Liu, Lina Gu, Yang Zheng, Meixiang Sang, Lingjiao Meng

**Affiliations:** ^1^ Research Center the Fourth Hospital of Hebei Medical University Shijiazhuang China; ^2^ The Third Department of Surgery the Fourth Hospital of Hebei Medical University Shijiazhuang China; ^3^ Hebei Key Laboratory of Precision Diagnosis and Comprehensive Treatment of Gastric Cancer the Fourth Hospital of Hebei Medical University Shijiazhuang China; ^4^ Tumor Research Institute the Fourth Hospital of Hebei Medical University Shijiazhuang China

**Keywords:** circ‐TNRC6B, circular RNA, DAG1, esophageal squamous cell carcinoma, miR‐452‐5p

## Abstract

Previous studies have uncovered the key role of circular RNAs (circRNAs) in various diseases, including cancer. However, the growth‐inhibitory effects of circRNAs on esophageal squamous cell carcinoma (ESCC) have not been completely elucidated. This study characterized a newly identified circRNA derived from exons 9–13 of TNRC6B (named circ‐TNRC6B). The expression of circ‐TNRC6B in ESCC tissues was markedly downregulated when compared to that in non‐tumor tissues. In 53 ESCC cases, circ‐TNRC6B expression was negatively correlated with the T stage. Multivariate Cox regression analysis showed that circ‐TNRC6B upregulation was an independent protective factor for ESCC patients' prognosis. Overexpression and knockdown functional experiments demonstrated that circ‐TNRC6B inhibited ESCC cell proliferation, migration, and invasion. RNA immunoprecipitation and dual‐luciferase reporter assays demonstrated that circ‐TNRC6B sponges oncogenic miR‐452‐5p to upregulate the expression and activity of DAG1. Treatment with miR‐452‐5p inhibitor partially reversed the circ‐TNRC6B‐induced changes in the biological behavior of ESCC cells. These findings demonstrated that circ‐TNRC6B exerts a tumor‐suppressing effect in ESCC through the miR‐452‐5p/DAG1 axis. Thus, circ‐TNRC6B is a potential prognostic biomarker for the clinical management of ESCC.

## Graphical abstract

This study identified a previously unknown tumor suppressor circular RNA called circ‐TNRC6B, which was significantly downregulated in esophageal squamous cell carcinoma (ESCC) tissues. circ‐TNRC6B downregulation predicted poor prognosis of patients with ESCC. Functionally, circ‐TNRC6B inhibited the proliferation, migration and invasion of ESCC cells. Mechanistically, circ‐TNRC6B sponged miR‐452‐5p to upregulate the downstream target gene *DAG1* in ESCC cells. The findings of this study suggest that circ‐TNRC6B may serve as a novel prognostic biomarker for ESCC.

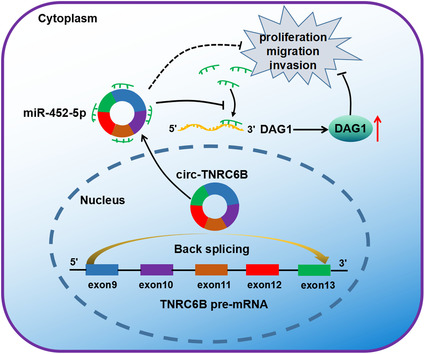

**Abbreviations**
ceRNA: competitive endogenous RNA
circRNAs: circular RNAs
DAG1: dystroglycan
EC: esophageal cancer
EMT: epithelial‐to‐mesenchymal transition
ESCC: esophageal squamous cell carcinoma
FISH: fluorescence *in situ* hybridization
gDNA: genomic DNA
Mbl: muscleblind
OS: overall survival
qRT‐PCR: quantitative reverse transcription PCR
RBPs: RNA‐binding proteins
RIP: RNA‐binding protein immunoprecipitation
RNase R: ribonuclease R
siRNA: short‐interfering RNA

## 1 Introduction

Esophageal cancer (EC), which is one of the most widespread digestive tract malignancies, is the seventh most common cause of cancer‐related death. The 5‐year survival rate of patients with EC is only 20% [1]. As the morbidity and mortality rates of EC in China are among the highest in the world, EC is a major health concern for the Chinese population [2]. Esophageal squamous cell carcinoma (ESCC), which mostly occurs in the middle or upper esophagus, is the predominant histological subtype of EC in China [3]. Although recent diagnostic and therapeutic advances have improved the survival of patients with EC, the improved survival rate is less pronounced at year three postdiagnosis in the elderly group and patients with ESCC [4]. Therefore, elucidating the molecular mechanisms of ESCC development will provide novel insights into the diagnosis, treatment, and prognosis of patients with ESCC.

Circular RNAs (circRNAs) are a special class of RNAs with closed loop structures without 5′ end caps and 3′ end poly(A) tails [5]. The unique circular structure of circRNAs contributes to their stable expression, resistance to ribonuclease R (RNase R) digestion, and long‐term transcriptional regulatory effects in cells [6]. Previous studies have demonstrated that circRNAs play a key role in carcinogenesis and that they are potential biomarkers for cancer diagnosis, treatment, and prognosis [7, 8, 9]. In ESCC, several circRNAs function as oncogenes to mediate tumorigenesis and tumor development. Zhou et al. reported that circPDE3B (annotated as hsa_circ_0000277 in circBase) promotes cell proliferation, migration, and invasion by inducing epithelial‐to‐mesenchymal transition (EMT) through the regulation of the miR‐4766‐5p/LAMA1 axis. This indicates that circPDE3B is a prognostic marker and a promising therapy target for ESCC [10]. CircGSK3β was reported to facilitate ESCC cell migration and invasion by directly interacting with GSK3β and inhibiting its activity, suggesting its potential diagnostic and prognostic values in ESCC [11]. The circRNA ciRS‐7 promotes the malignant progression of ESCC by modulating the miR‐7/HOXB13 or miR‐876‐5p/MAGE‐A family axis [12, 13]. Additionally, mitochondrial circPUM1 serves as a scaffold for the interaction between UQCRC1 and UQCRC2 and consequently stabilizes mitochondrial complex III, enhancing oxidative phosphorylation and inhibiting pyroptosis to promote ESCC cell proliferation during malignancy [14].

A subset of circRNAs also functions as tumor suppressors during ESCC progression. For instance, circFAM120B was reported to suppress proliferation and invasion through the miR‐661/PPM1L and PKR/p38 MAPK/EMT pathways, suggesting its potential as a biomarker for ESCC [15]. Previously, we demonstrated that circ‐DOCK5 inhibits the metastasis of ESCC by modulating the miR‐627‐3p/TGFB2 signaling pathway [16]. The *CNTNAP3*‐derived circRNA cCNTNAP3 inhibits cell proliferation and promotes apoptosis in ESCC cells expressing wild‐type TP53 by sponging miR‐513a‐5p. Thus, targeting cCNTNAP3 is a potential novel therapeutic strategy for ESCC [17]. Circ‐SMAD7 inhibits tumor cell proliferation and migration during ESCC progression, indicating its potential as a biomarker for ESCC diagnosis and therapy [18]. Additionally, the *FOXO3*‐derived circRNA circ‐FOXO3 inhibits ESCC cell growth, migration, and invasion through the miR‐23a/PTEN axis. Thus, circ‐FOXO3 is a potential therapeutic target for ESCC [19].

This study identified a tumor suppressor circRNA derived from exons 9–13 of TNRC6B (circBase ID: hsa_circ_0063411) (called circ‐TNRC6B). The expression of circ‐TNRC6B in ESCC tissues was markedly downregulated when compared to that in non‐tumor tissues. Clinical data analysis showed that circ‐TNRC6B expression was negatively correlated with the T stage. Thus, circ‐TNRC6B can serve as an independent prognostic factor for patients with ESCC. Functionally, circ‐TNRC6B inhibited ESCC cell proliferation, migration, and invasion by sponging miR‐452‐5p to upregulate DAG1 expression. Our findings demonstrated that circ‐TNRC6B is a potential prognostic biomarker for ESCC.

## 2 Materials and methods

### 2.1 Clinical tissue chip array samples

An organized chip array of 53 ESCC tissues and 48 non‐tumor tissues (HEsoS105Su01, XT17‐018) was purchased from Outdo Biotech Corporation (Shanghai, China). The clinicopathological characteristics and survival status of patients were obtained from follow‐up data. All patients involved in this study signed written informed consent statements and accepted the protocol approved by the Institutional Review Board of Shanghai Outdo Biotech Corporation (license number: SHYJS‐CP‐1707008). The study methodologies conformed to the standards set by the Declaration of Helsinki.

### 2.2 Cell culture

The human ESCC cell lines TE‐1 (RRID:CVCL_1759), KYSE‐30 (RRID:CVCL_1351), KYSE‐150 (RRID:CVCL_1348), and KYSE‐170 (RRID:CVCL_1358) were purchased from Procell Life Science & Technology Co., Ltd (Wuhan, China) and stored in our laboratory. These cell lines have been authenticated in the past 3 years using short tandem repeat analysis. TE‐1, KYSE‐30, and KYSE‐150 cells were cultured in Roswell Park Memorial Institute (RPMI) 1640 medium, whereas KYSE‐170 cells were cultured in Dulbecco's modified Eagle's medium (DMEM) supplemented with 10% heat‐inactivated FBS (GIBCO, Thornton, NSW, Australia) in a 37 °C sterile incubator with 5% CO_2_. In this study, the cells used in all experiments were mycoplasma‐free.

### 2.3 DNA/RNA extraction and agarose gel electrophoresis

The genomic DNA (gDNA) was extracted from the ESCC cell lines KYSE150 and KYSE170 using the DNA extraction kit (TIANGEN, Beijing, China), following the manufacturer's instructions. Total RNA was extracted from ESCC cells using TRIzol reagent (Invitrogen, Frederick, MA, USA). Reverse transcription polymerase chain reaction (PCR) analysis was performed using GoTaq® Green Master Mix (Promega, Madison, WI, USA). The amplicons were resolved using a 2% agarose gel. The primers used in the reaction are listed in Table S1. The grayscale of nucleic acid bands was detected using ultraviolet irradiation.

### 2.4 Quantitative real‐time PCR (qRT‐PCR), nuclear and cytoplasmic fractionation, and actinomycin D treatment

Total RNA was reverse‐transcribed into cDNA using the GoScript Reverse Transcription System kit (Promega), following the manufacturer's instructions. PCR amplification was performed using GoTaq qPCR Master Mix (Promega). The primers used in the amplification procedure are listed in Table S1. Bulge‐loop miRNA RT and forward primer specific for miR‐452‐5p were purchased from RiboBio Company (Guangzhou, China). The relative expression levels of target genes were determined using the $2^{-\Delta \Delta C_{T}}$ method. Nuclear and cytoplasmic fractions were prepared using the Thermo Scientific NE‐PER nuclear and cytoplasmic extraction kit (ThermoFisherPierce Biotechnology, Rockford, IL, USA), following the manufacturer's instructions. Briefly, cells were incubated in cell fractionation buffer for 5 min on ice and centrifuged. The cytoplasmic fraction and nuclear pellet were separated. The samples were incubated with lysis/binding solution at 25 °C to split the nuclear and cytoplasmic fractions. The cytoplasmic and nuclear RNA fractions were isolated using a filter cartridge and eluted using an elution solution. The samples were subjected to qRT‐PCR analysis. The cells were treated with actinomycin D (2.0 μg·mL^−1^) to detect the stability of circ‐TNRC6B and its related linear mRNA.

### 2.5 Plasmid construction

To construct the circ‐TNRC6B overexpression plasmid, hsa_circ_0063411 sequence was amplified and cloned into the pLC5‐ciR empty vector (Geneseed Biotech Corporation, Guangzhou, China). The wild‐type sequence of hsa_circ_0063411 was synthesized and cloned into psiCHECK‐2 empty vector (Geneseed Biotech Corporation) to generate the circ‐TNRC6B luciferase reporter plasmid. DAG1 3′‐UTR plasmid was constructed by synthesizing the wild‐type or mutant sequence of DAG1 3′‐UTR and cloning it into the psiCHECK‐2 empty vector (Geneseed Biotech Corporation).

### 2.6 Fluorescence *in situ* hybridization

The biotin‐labeled circ‐TNRC6B probe was purchased from Geneseed Biotech Corporation. The 5′ carboxyfluorescein (5′ FAM)‐modified miR‐452‐5p probe was designed and synthesized by GenePharma Corporation (Shanghai, China). The probe sequences are shown in Table S1. To perform cellular fluorescence *in situ* hybridization (FISH), KYSE150, and KYSE170 cells were seeded on coverslips and cultured for 12 h. The cells were rinsed with PBS and fixed with 4% paraformaldehyde. Next, the samples were treated with 0.2% Triton X‐100 on ice. The permeabilized samples were incubated with a blocking solution to block the nonspecific sites and hybridized with the probes for 12 h at 37 °C. The fluorescence signal of circ‐TNRC6B was detected with Cy5‐streptavidin conjugate (Invitrogen). The nuclei were counterstained with 4′,6‐diamidino‐2‐phenylindole (DAPI) for 10 min. To perform tissue FISH, ESCC chip array tissue slides were deparaffinized in xylene and ethanol solutions, followed by treatment with proteinase K at 37 °C for 10 min before blocking. The subsequent procedures were similar to those used in the cellular FISH analysis. The images were snapped using an LSM 900 confocal microscope (ZEISS, Oberkochen, Germany).

### 2.7 Cell counting kit‐8 assay

Esophageal squamous cell carcinoma cells belonging to different treatment groups were seeded in a 96‐well plate (2000 cells per well). After cell attachment, cell proliferation viability was examined using cell counting kit‐8 (CCK‐8) reagent (MCE, MedChemExpress, shanghai, China) once every 24 h for 5 days. Briefly, the cells were incubated with 10 μL CCK‐8 reagent in a 37 °C incubator for 2 h. The absorbance of the reaction mixture at 450 nm was measured using a microplate reader (Tecan, Männedorf, Switzerland).

### 2.8 Colony formation assay

Esophageal squamous cell carcinoma cells belonging to different treatment groups were planted in a 6‐well plate (2000 cells per well) and incubated for 2 weeks with the complete medium. First, the cells were treated with 4% paraformaldehyde for fixation. After rinsing with PBS, the cells were stained with crystal violet. Finally, the cell colonies were counted and imaged.

### 2.9 Wound healing assay

Esophageal squamous cell carcinoma cells belonging to different treatment groups were planted in a 6‐well plate and incubated for 12–24 h until the cell convergence reached 80%. The cell monolayer was scratched with a pipette tip. The cells were washed three times and incubated with the fresh serum‐free medium. The images of the monolayer were captured under a microscope at 0 and 24 h. The scratch healing rate was evaluated by examining the migration distance as follows: Scratch healing rate (%) = [(scratch width at 0 h − scratch width at 24 h)/(scratch width at 0 h)] × 100%.

### 2.10 Transwell migration and invasion assay

Cell migration and invasion abilities were investigated with transwell compartment. For migration analysis, cells belonging to different treatment groups (5 × 10^4^) were resuspended in the serum‐free medium and cultured in the upper chamber for 24–48 h. The lower chamber was filled with the culture medium supplemented with 10% FBS. For invasion analysis, Matrigel gel (BD Biosciences, Franklin Lakes, NJ, USA) was evenly added to the bottom of the chamber at 37 °C for 12 h. The subsequent procedures were analogous to migration analysis. The cells were fixed with 4% paraformaldehyde and stained with crystal violet. The number of cells that migrated or invaded through the membranes was counted under a microscope.

### 2.11 Dual‐luciferase reporter gene assay

KYSE150 cells were cultured in 12‐well plates and co‐transfected with luc‐empty vector, wild‐type or mutant luc‐circ‐TNRC6B vector, and negative control (NC) or miR‐452‐5p mimics, wild‐type or mutant DAG1 3′‐UTR, and NC or miR‐452‐5p mimics using Hiperfect transfection reagent. The cell lysates were prepared using the dual‐luciferase reporter gene assay kit (Beyotime, Shanghai, China), following the manufacturer's instructions. The luciferase activities were measured using Spark Microplate Reader (Tecan, Männedorf, Switzerland). Luciferase activity was calculated as the ratio of firefly luciferase activity to Renilla luciferase activity.

### 2.12 RNA immunoprecipitation

KYSE150 cells were co‐transfected with Myc‐AGO2 vector or Myc‐empty vector and NC or miR‐452‐5p mimics for 48 h. The samples were subjected to RNA immunoprecipitation (RIP) using the Magna RIP kit (Millipore, Darmstadt, Germany), following the manufacturer's instructions. Briefly, the cells were lysed with RIP lysis buffer for 5 min. The supernatant was incubated with immunoprecipitation buffer containing 0.5 m ethylenediaminetetraacetic acid and RNase inhibitor. The prepared cell lysates were incubated with anti‐Myc or IgG control antibodies coupled to magnetic beads at 4 °C for 12 h. The beads were eluted with cold RIP wash buffer and incubated with protease K buffer at 55 °C for 30 min. The supernatant was collected via centrifugation and incubated with TRIzol reagent to extract the immunoprecipated RNA. The RNA samples were purified and subjected to qRT‐PCR analysis.

### 2.13 Western blotting

Esophageal squamous cell carcinoma cells were harvested and centrifuged at low temperature. The cells were lysed using radioimmunoprecipitation assay lysis buffer containing 1% phenylmethylsulfonyl fluoride to isolate total proteins. After protein quantification, equal amounts of denatured proteins (50 μg) were subjected to SDS/‐PAGE with a 10% gel. The resolved proteins were transferred to a polyvinylidene difluoride (PVDF) membrane. The membrane was incubated with rabbit anti‐DAG1 polyclonal antibodies (11017‐1‐AP; Proteintech) and anti‐ACTB antibodies (20536‐1‐AP; Proteintech, Rosemont, IL, USA) at 4 °C for 12 h. Next, the membrane was incubated with horseradish peroxidase‐conjugated Affinipure goat anti‐rabbit IgG (H + L) (SA00001‐2; Proteintech) at 37 °C for 1 h. The membrane was then washed with Tris‐buffered saline containing Tween‐20 (TBST). Immunoreactive signals were developed using enhanced chemiluminescence plus reagents (Solarbio, Beijing, China).

### 2.14 Statistical analysis

All statistical analyses were performed using spss 26.0 (IBM, Chicago, USA) and graphpad prism 8.0 software (San Diego, CA, USA). Data are represented as mean ± standard deviation (SD). Means between different groups were compared using Mann–Whitney *U*‐test and analysis of variance (ANOVA). Categorical data were compared using the chi‐squared test. Univariate and multivariate Cox regression analyses were performed to evaluate the factors associated with the overall survival (OS) of patients with ESCC. Kaplan–Meier survival curves were compared using the log‐rank test to analyze the effect of clinicopathological variables and circRNA expression on the OS of patients. All statistical tests were two‐tailed. Differences were considered significant at *P* < 0.05.

## 3 Results

### 3.1 Characterization of circ‐TNRC6B in ESCC cells

**Fig. 1:**
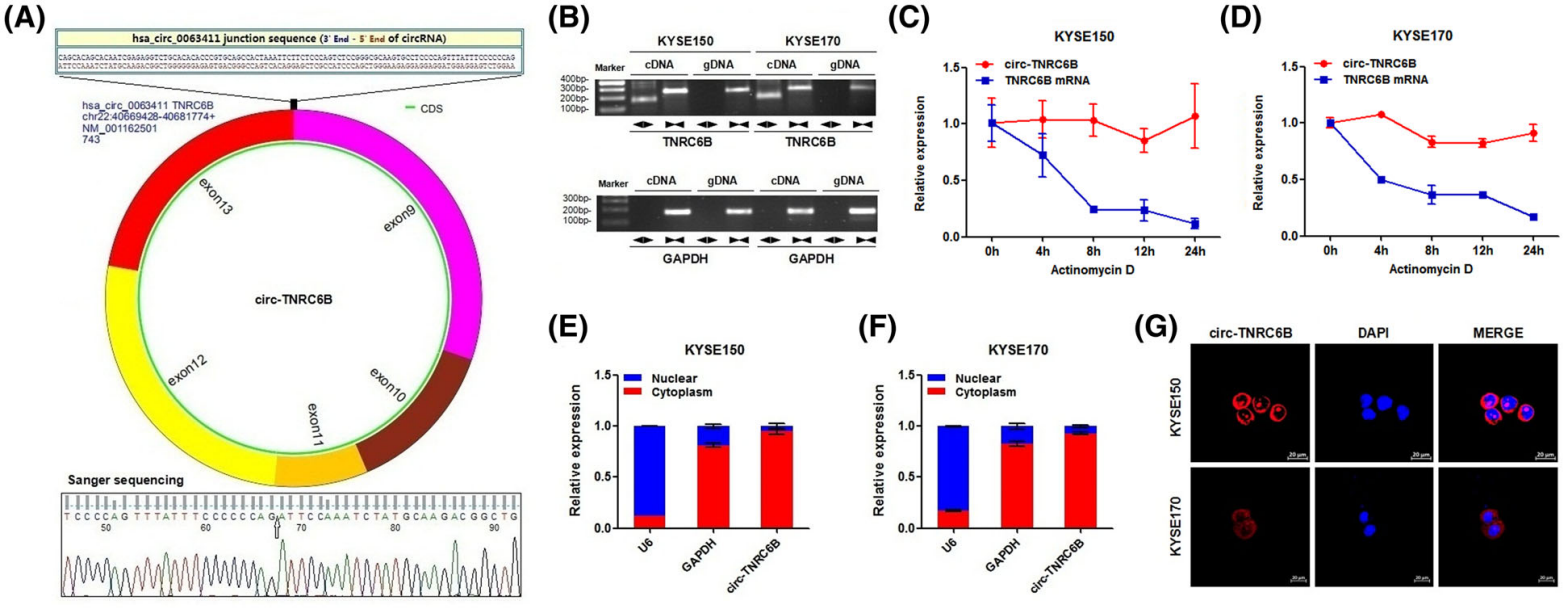
Characterization of circ‐TNRC6B in ESCC cells. (A) Schematic diagram of the cyclization of circ‐TNRC6B (circBase ID: hsa_circ_0063411) based on circBase database and circprimer 2.0 software. The back‐spliced junction sequences of circ‐TNRC6B were analyzed by CircInteractome database and verified by Sanger sequencing with divergent primers. (B) The existence of circ‐TNRC6B was validated in KYSE150 and KYSE170 cells by RT‐PCR. Divergent primers amplified circ‐TNRC6B in cDNA samples but not gDNA samples, while convergent primers amplified TNRC6B linear transcript in both cDNA and gDNA samples. GAPDH was selected as the reference control group (*n* = 2). (C, D) The relative expression levels of circ‐TNRC6B and TNRC6B mRNA were detected by qRT‐PCR after treatment with Actinomycin D in KYSE150 and KYSE170 cells at the indicated time points. Data are represented as mean ± SD (*n* = 2). (E, F) The cellular localization of circ‐TNRC6B was evaluated by nuclear and cytoplasmic fractionation assay in KYSE150 and KYSE170 cells. U6 and GAPDH were selected as nuclear and cytoplasmic control groups, respectively. Data are represented as mean ± SD (*n* = 2). (G) The cellular localization of circ‐TNRC6B was evaluated by FISH analysis in KYSE150 and KYSE170 cells (*n* = 3). Nuclei were stained with DAPI solution. Scale bar: 20 μm.

According to circBase database and circprimer 2.0 software [20, 21], circ‐TNRC6B (circBase ID: hsa_circ_0063411) originates from exons 9–13 of TNRC6B located on chromosome 22 (40669428–40681774) in the human genome and has a length of 743 nt (Fig. 1A). The back‐spliced junction sequences of circ‐TNRC6B were analyzed using the CircInteractome database [22] and verified using Sanger sequencing with divergent primers (Fig. 1A). qRT‐PCR analysis revealed that circ‐TNRC6B can only be amplified from cDNA samples with divergent primers but not from gDNA samples of KYSE150 and KYSE170 cells (Fig. 1B). The half‐life of circ‐TNRC6B within 24 h was longer than that of linear TNRC6B mRNA transcript in KYSE150 and KYSE170 cells treated with actinomycin D (Fig. 1C,D). The results of the nuclear and cytoplasmic fractionation assay with KYSE150 and KYSE170 cells revealed that circ‐TNRC6B was predominantly distributed in the cytoplasm (Fig. 1E,F), which was accordant with the FISH analysis results (Fig. 1G; Fig. S1). These data indicate that circ‐TNRC6B is a bona fide and stable transcript in ESCC cells.

### 3.2 circ‐TNRC6B is downregulated in ESCC tissues and circ‐TNRC6B downregulation predicts poor prognosis of ESCC patients

**Fig. 2:**
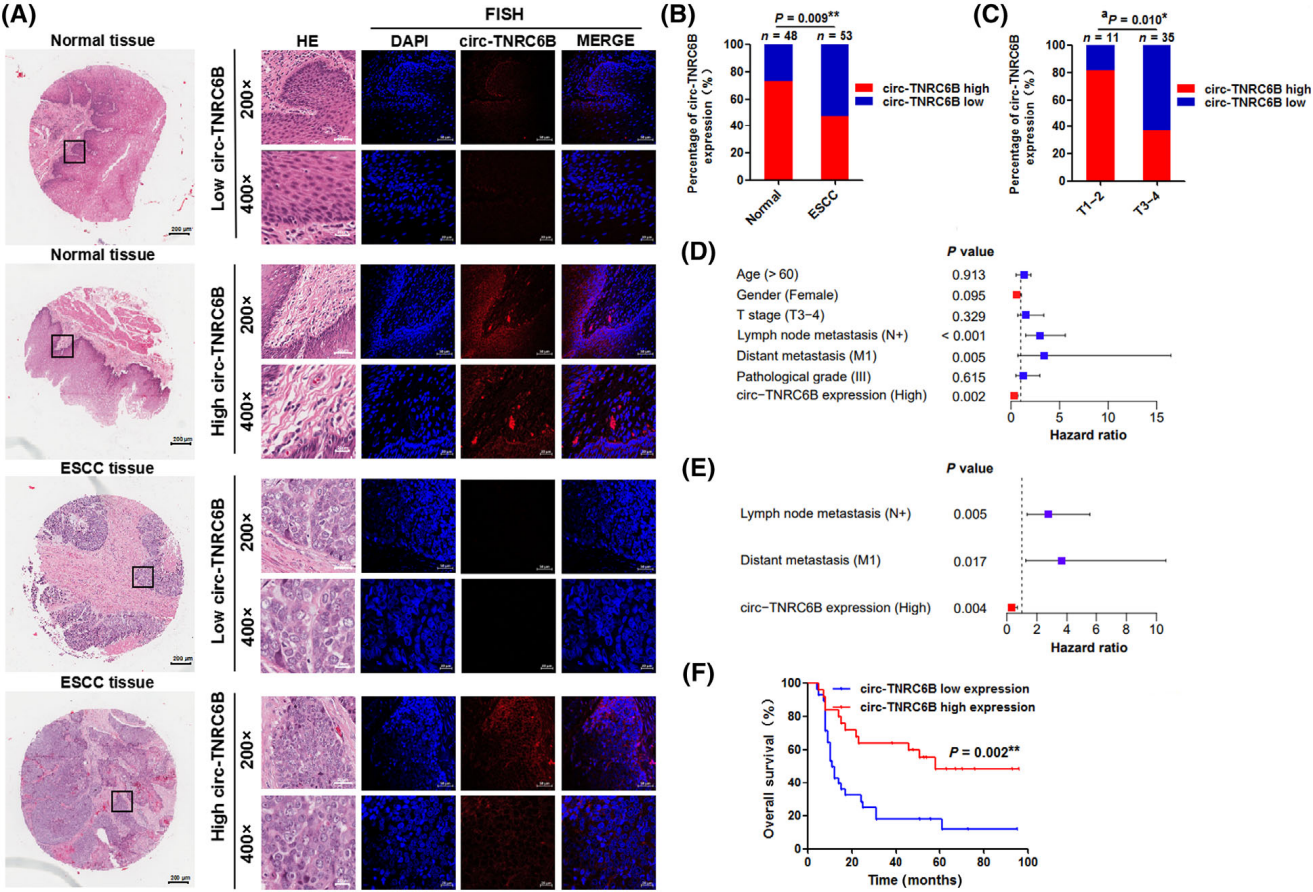
circ‐TNRC6B is downregulated in ESCC tissues and its low expression predicts poor prognosis in ESCC patients. (A) The representative images of circ‐TNRC6B expression in ESCC tissues and adjacent normal tissues by FISH assay (*n* = 3). Scale bar: 200 μm (whole tissue image), 50 μm (200× image), and 20 μm (400× image). (B) The percentage of circ‐TNRC6B expression in 53 ESCC tissues and 48 adjacent normal tissues. *P*‐values were determined by chi‐squared test. (C) The percentage of T stage in 25 ESCC tissues with circ‐TNRC6B high expression and 28 ESCC tissues with circ‐TNRC6B low expression. *P*‐values were determined by chi‐squared test. (D, E) Univariate and multivariate Cox regression analyses of prognostic factors for ESCC patients. The bars indicate 95% CI. (F) Kaplan–Meier curve for OS rate according to circ‐TNRC6B expression, *n* = 25 (circ‐TNRC6B high expression), 28 (circ‐TNRC6B low expression). Survival analyses were performed by log‐rank test. *P < 0.05, **P < 0.01. ^a^Numbers do not equal to the total number due to missing data.

To investigate the expression profile of circ‐TNRC6B in ESCC tissues and its correlation with the clinical features and prognosis of ESCC patients, the expression level of circ‐TNRC6B in a chip array of 53 ESCC tissues and 48 non‐tumor tissues was examined using FISH analysis. The representative images of circ‐TNRC6B expression in ESCC tissues and non‐tumor tissues are shown in Fig. 2A. The expression of circ‐TNRC6B in ESCC tissues was downregulated when compared with that in non‐tumor tissues (Fig. 2B). These results were consistent with those of RNA sequencing of 73 paired ESCC and non‐tumor tissues (Fig. S2) [23]. The expression level of circ‐TNRC6B was significantly and negatively correlated with T stage (Fig. 2C; Table S2) but was not significantly correlated with age, gender, lymph node metastasis, distant metastasis, and pathological grade (Fig. S3A–E, Table S2). Next, the effect of circ‐TNRC6B expression on the prognosis of patients with ESCC was examined. The OS of patients according to the expression of circ‐TNRC6B is shown in Fig. S3F,G. Univariate analysis indicated that circ‐TNRC6B expression, lymph node metastasis, and distant metastasis were significantly correlated with OS (Fig. 2D; Table S3). Multivariate analysis revealed that lymph node metastasis and distant metastasis were independent risk factors for OS, while circ‐TNRC6B expression was an independent protective factor for OS in patients with ESCC (Fig. 2E; Table S3). Kaplan–Meier survival curve analysis suggested that circ‐TNRC6B downregulation, positive lymph node metastasis, and distant metastasis were associated with decreased OS (Fig. 2F; Fig. S3H,I). However, age, gender, T stage, and pathological grade were not significantly correlated with OS in these 53 patients with ESCC (Fig. S3J–M). These data suggest that circ‐TNRC6B downregulation is correlated with ESCC progression and that circ‐TNRC6B may serve as an independent prognostic factor for patients with ESCC.

### 3.3 circ‐TNRC6B suppresses the proliferation, migration, and invasion of ESCC cells

**Fig. 3:**
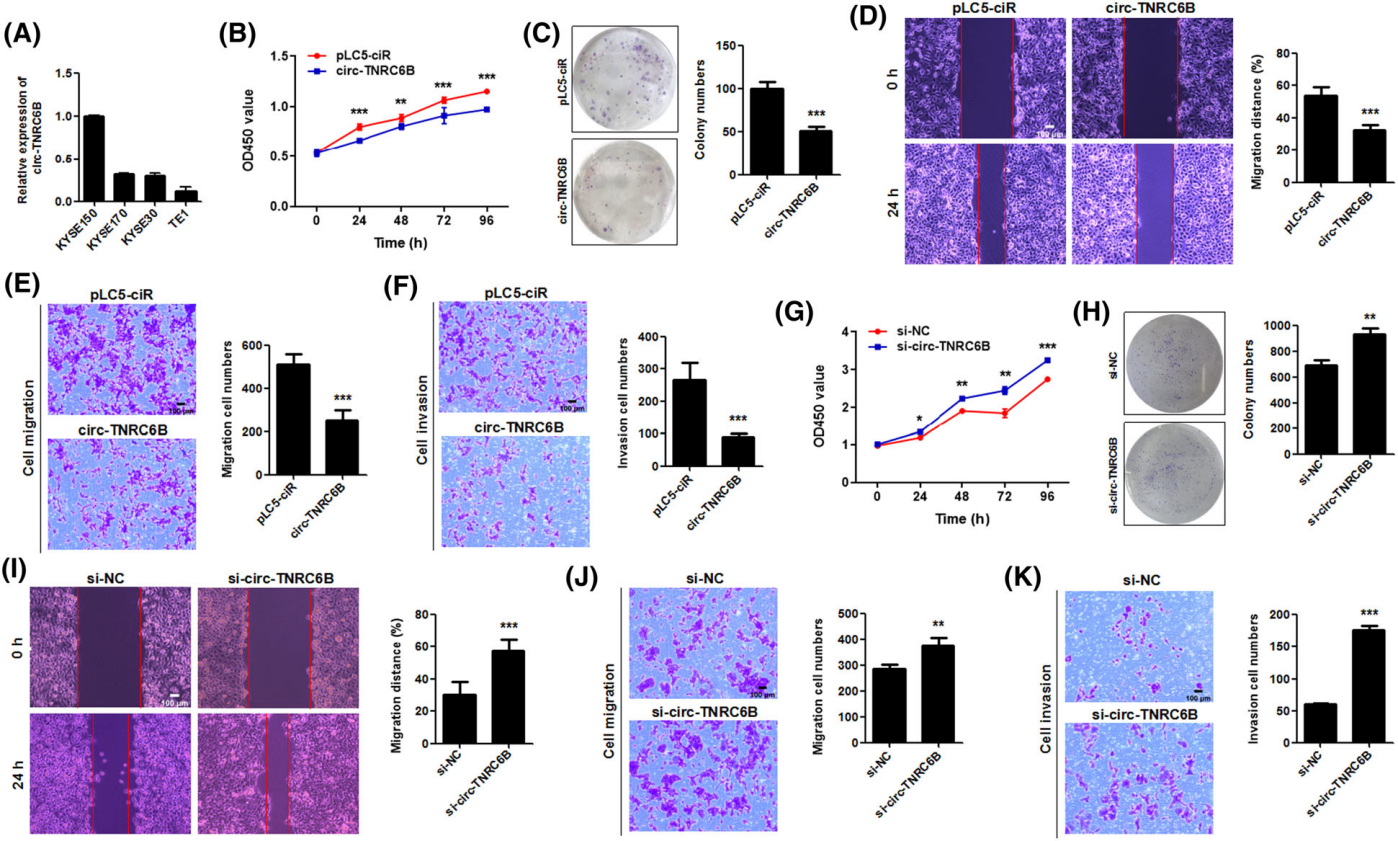
circ‐TNRC6B suppresses the proliferation, migration, and invasion abilities of ESCC cells. (A) The relative expression level of circ‐TNRC6B in four ESCC cell lines was examined by qRT‐PCR (*n* = 2). (B, C). The proliferative and clonogenic ability of TE1 cells after exogenous circ‐TNRC6B transfection was detected by CCK‐8 (*n* = 4, *P*‐values were determined by two‐way ANOVA) and colony formation assay (*n* = 3, *P*‐values were determined by Mann–Whitney *U*‐test). Data are represented as mean ± SD. (D, E) The migration ability of TE1 cells after exogenous circ‐TNRC6B transfection was detected by wound healing and transwell migration assay. Data are represented as mean ± SD (*n* = 3, *P*‐values were determined by Mann–Whitney *U*‐test). Scale bar: 100 μm. (F) The invasion ability of TE1 cells after exogenous circ‐TNRC6B transfection was detected by transwell invasion assay. Data are represented as mean ± SD (*n* = 3, *P*‐values were determined by Mann–Whitney *U*‐test). Scale bar: 100 μm. (G, H) The proliferative and clonogenic ability of KYSE150 cells after silencing circ‐TNRC6B expression was detected by CCK‐8 (*n* = 4, *P*‐values were determined by two‐way ANOVA) and colony formation assay (*n* = 3, *P*‐values were determined by Mann–Whitney *U*‐test). si‐NC: negative control of siRNA. (I, J) The migration ability of KYSE150 cells after silencing circ‐TNRC6B expression was detected by wound healing and transwell migration assay. Data are represented as mean ± SD (*n* = 3, *P*‐values were determined by Mann–Whitney *U*‐test). Scale bar: 100 μm. (K) The invasion ability of KYSE150 cells after silencing circ‐TNRC6B expression was detected by transwell invasion assay. Data are represented as mean ± SD (*n* = 3, *P*‐values were determined by Mann–Whitney *U*‐test). Scale bar: 100 μm. *P < 0.05, **P < 0.01, ***P < 0.001.

To explore the biological effects of circ‐TNRC6B, the expression level of circ‐TNRC6B in four ESCC cell lines was examined using qRT‐PCR. The endogenous expression of circ‐TNRC6B was the lowest in TE1 cells and the highest in KYSE150 cells (Fig. 3A). Therefore, TE1 cells were selected for overexpressing exogenous circ‐TNRC6B, while KYSE150 cells were selected for silencing endogenous circ‐TNRC6B. The results of the CCK‐8 and colony formation assay revealed that the proliferative and clonogenic ability of TE1 cells was significantly decreased after exogenous circ‐TNRC6B transfection (Fig. 3B,C). Meanwhile, the results of the wound healing and transwell assay indicated the migration and invasion of TE1 cells were significantly suppressed upon transfection with exogenous circ‐TNRC6B (Fig. 3D–F). KYSE150 cells were transfected with circ‐TNRC6B splice junction‐specific short‐interfering RNA (siRNA) to downregulate endogenous circ‐TNRC6B expression. CCK‐8 and colony formation assay results demonstrated that circ‐TNRC6B knockdown significantly enhanced the proliferative and clonogenic ability of KYSE150 cells (Fig. 3G,H). Meanwhile, the results of the wound healing and transwell assays indicated circ‐TNRC6B knockdown significantly enhanced the migration and invasion of KYSE150 cells (Fig. 3I–K). These data suggested that circ‐TNRC6B functions as a tumor suppressor to inhibit the proliferation, migration, and invasion of ESCC cells.

### 3.4 circ‐TNRC6B functions as a miR‐452‐5p sponge in ESCC cells

**Fig. 4:**
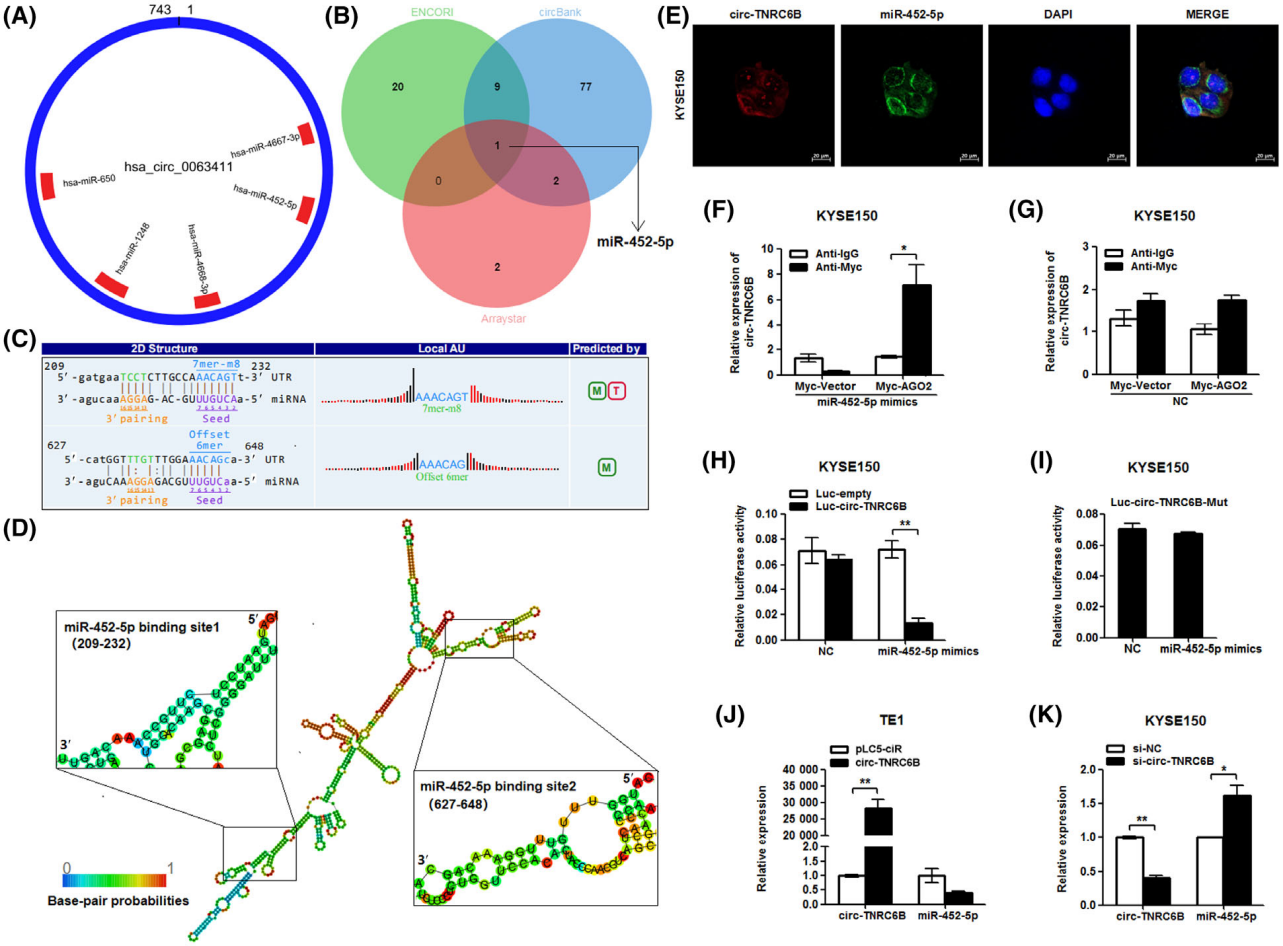
circ‐TNRC6B serves as a miR‐452‐5p sponge in ESCC cells. (A) CircRNA‐miRNA binding circle diagram showing potential miRNA targets of circ‐TNRC6B predicted by Arraystar's home‐made miRNA target prediction software based on TargetScan and miRanda. (B) Venn diagram showing the intersection of potential miRNA targets of circ‐TNRC6B based on Arraystar's home‐made miRNA target prediction software, ENCORI, and circBank databases. (C) The complementary sequences of circ‐TNRC6B and miR‐452‐5p were predicted by Arraystar's home‐made miRNA target prediction software. (D) The MFE secondary structure of circ‐TNRC6B predicted by RNAfold web server and the part where the secondary structure of circ‐TNRC6B is relevant for the binding of miR‐452‐5p. (E) FISH assay indicated circ‐TNRC6B and miR‐452‐5p were co‐localized in the cytoplasm of KYSE150 cells (*n* = 3). Scale bar: 20 μm. (F) RIP analysis in KYSE150 cells co‐transfected with Myc‐AGO2 vector and miR‐452‐5p mimics. Data are represented as mean ± SD (*n* = 3, *P*‐values were determined by Mann–Whitney *U*‐test). (G) RIP analysis in KYSE150 cells co‐transfected with Myc‐AGO2 vector and NC (*n* = 2, *P*‐values were determined by Mann–Whitney *U*‐test). Data are represented as mean ± SD. (H) Dual‐luciferase reporter gene analysis detected the luciferase activity of circ‐TNRC6B when co‐transfected with luc‐empty vector or wild‐type luc‐circ‐TNRC6B vector and NC or miR‐452‐5p mimics in KYSE150 cells. Data are represented as mean ± SD (*n* = 3, *P*‐values were determined by Mann–Whitney *U*‐test). (I) Dual‐luciferase reporter gene analysis detected the luciferase activity of circ‐TNRC6B when co‐transfected with mutant luc‐circ‐TNRC6B vector and NC or miR‐452‐5p mimics in KYSE150 cells. Data are represented as mean ± SD (*n* = 3, *P*‐values were determined by Mann–Whitney *U*‐test). (J) TE1 cells were transfected with pLC5 empty vector or circ‐TNRC6B vector; then, the relative expression of circ‐TNRC6B and miR‐452‐5p were detected by qRT‐PCR assay. Data are represented as mean ± SD (*n* = 2, *P*‐values were determined by Mann–Whitney *U*‐test). (K) KYSE150 cells were transfected with si‐NC or si‐circ‐TNRC6B; then, the relative expression of circ‐TNRC6B and miR‐452‐5p was detected by qRT‐PCR assay. Data are represented as mean ± SD (*n* = 2, *P*‐values were determined by Mann–Whitney *U*‐test). *P < 0.05, **P < 0.01.

As circ‐TNRC6B is mainly distributed in the cytoplasm, its ability to function as a miRNA sponge in ESCC cells was examined. Analysis of data with home‐made miRNA target prediction software from Arraystar (Rockville, MA, USA) based on TargetScan and miRanda data revealed that circ‐TNRC6B may bind to hsa‐miR‐650, hsa‐miR‐1248, hsa‐miR‐4668‐3p, hsa‐miR‐452‐5p, and hsa‐miR‐4667‐3p (Fig. 4A). Data from ENCORI and circBank databases were retrieved to predict miRNAs that can interact with circ‐TNRC6B [24, 25]. The results were compared with the miRNAs predicted by home‐made miRNA target prediction software from Arraystar. miR‐452‐5p was selected as a candidate miRNA for further analysis (Fig. 4B). The secondary structure of circRNA affects its ability to sponge miRNA [26]. This study predicted the secondary structure of circ‐TNRC6B using the RNAfold web server (Fig. S4). The complementary sequences of circ‐TNRC6B and miR‐452‐5p were predicted using home‐made miRNA target prediction software from Arraystar (Fig. 4C). The region in the secondary structure of circ‐TNRC6B relevant for the binding of miR‐452‐5p is shown in Fig. 4D. FISH analysis indicated that circ‐TNRC6B and miR‐452‐5p were co‐localized to the cytoplasm in KYSE150 cells (Fig. 4E). RIP analysis demonstrated that circ‐TNRC6B was significantly enriched with anti‐Myc antibodies in KYSE150 cells co‐transfected with Myc‐AGO2 vector and miR‐452‐5p mimics (Fig. 4F). However, co‐transfection with Myc‐AGO2 vector and NC did not affect the enrichment of circ‐TNRC6B (Fig. 4G). The results of the dual‐luciferase reporter assay suggested that miR‐452‐5p overexpression markedly downregulated the luciferase activity of wild‐type luc‐circ‐TNRC6B in KYSE150 cells (Fig. 4H), while there was no significant change when miR‐452‐5p was co‐transfected with mutant luc‐circ‐TNRC6B vector (Fig. 4I). Moreover, circ‐TNRC6B overexpression downregulated the expression of miR‐452‐5p in TE1 cells (Fig. 4J). In contrast, circ‐TNRC6B knockdown significantly upregulated the expression of miR‐452‐5p in KYSE150 cells (Fig. 4K). Together, the above findings support our hypothesis that circ‐TNRC6B sponges miR‐452‐5p in ESCC cells.

### 3.5 miR‐452‐5p promotes the proliferation, migration, and invasion of ESCC cells

**Fig. 5:**
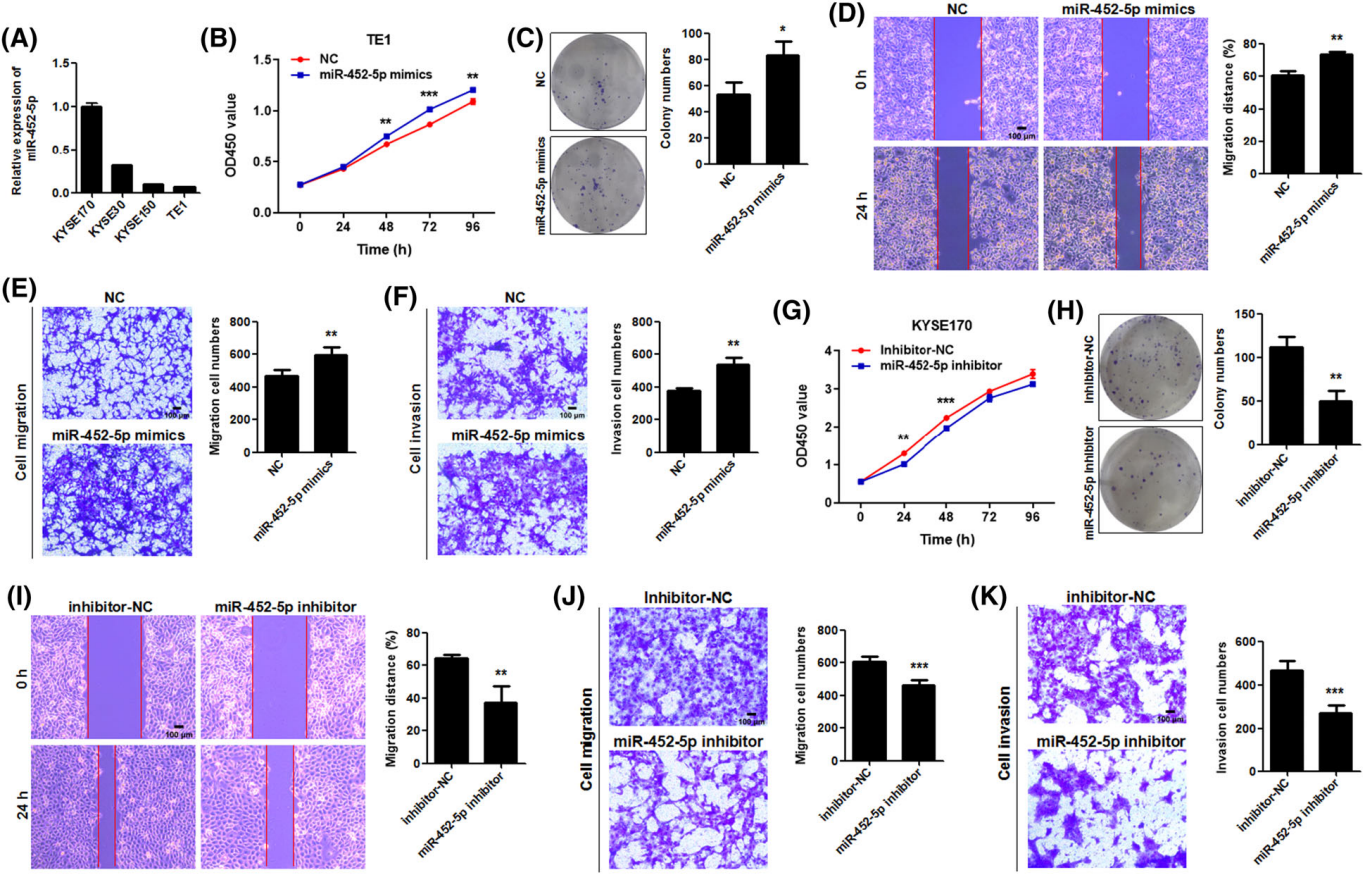
miR‐452‐5p promotes the proliferation, migration, and invasion abilities of ESCC cells. (A) The relative expression level of miR‐452‐5p in four ESCC cell lines was examined by qRT‐PCR (*n* = 2). Data are represented as mean ± SD. (B, C) The proliferative and clonogenic ability of TE1 cells after exogenous miR‐452‐5p mimics transfection was detected by CCK‐8 (*n* = 4, *P*‐values were determined by two‐way ANOVA) and colony formation assay (*n* = 3, *P*‐values were determined by Mann–Whitney *U*‐test). NC: negative control of miRNA mimics. Data are represented as mean ± SD. (D, E) The migration ability of TE1 cells after exogenous miR‐452‐5p mimics transfection was detected by wound healing and transwell migration assay. Data are represented as mean ± SD (*n* = 3, *P*‐values were determined by Mann–Whitney *U*‐test). Scale bar: 100 μm. (F) The invasion ability of TE1 cells after exogenous miR‐452‐5p mimics transfection was detected by transwell invasion assay. Data are represented as mean ± SD (*n* = 3, *P*‐values were determined by Mann–Whitney *U*‐test). Scale bar: 100 μm. (G, H) The proliferative and clonogenic ability of KYSE170 cells after miR‐452‐5p inhibitor transfection was detected by CCK‐8 (*n* = 4, *P*‐values were determined by two‐way ANOVA) and colony formation assay (*n* = 3, *P*‐values were determined by Mann–Whitney *U*‐test). Inhibitor NC: negative control of miRNA inhibitor. Data are represented as mean ± SD. (I, J) The migration ability of KYSE170 cells after miR‐452‐5p inhibitor transfection was detected by wound‐healing and transwell migration assay. Data are represented as mean ± SD (*n* = 3, *P*‐values were determined by Mann–Whitney *U*‐test). Scale bar: 100 μm. (K) The invasion ability of KYSE170 cells after miR‐452‐5p inhibitor transfection was detected by transwell invasion assay. Data are represented as mean ± SD (*n* = 3, *P*‐values were determined by Mann–Whitney *U*‐test). Scale bar: 100 μm. *P < 0.05, **P < 0.01, ***P < 0.001.

Based on ENCORI pan‐cancer data integrated from The Cancer Genome Atlas project, miR‐452‐5p expression in esophageal carcinoma tissues was significantly higher than that in non‐tumorous esophageal tissues (Fig. S5A). We next investigated the effect of miR‐452‐5p on the biological behavior of ESCC cells. The expression level of miR‐452‐5p in four ESCC cell lines was evaluated using qRT‐PCR. The results showed miR‐452‐5p expression was relatively low in TE1 and KYSE150 cells, while KYSE170 cells expressed the highest levels of miR‐452‐5p among cell lines tested (Fig. 5A). Hence, TE1 and KYSE150 cells were selected for miR‐452‐5p overexpression experiments. The results of the CCK‐8 and colony formation assay demonstrated that transfection with miR‐452‐5p mimics markedly upregulated the proliferative and clonogenic ability of TE1 cells (Fig. 5B,C). Similar results were obtained with KYSE150 cells (Fig. S5B,C). Wound‐healing assay, transwell migration, and invasion analysis indicated that miR‐452‐5p mimics enhanced the migration and invasion of TE1 (Fig. 5D–F) and KYSE150 cells (Fig. S5D–F). KYSE170 cells were transfected with miR‐452‐5p inhibitor for interference experiments. CCK‐8 and colony formation assay demonstrated that transfection with miR‐452‐5p inhibitor markedly decreased the proliferative and clonogenic ability of KYSE170 cells (Fig. 5G,H). Meanwhile, wound‐healing assay, transwell migration, and invasion analysis demonstrated that miR‐452‐5p inhibitor suppressed the migration and invasion of KYSE170 cells (Fig. 5I–K). These findings suggest that miR‐452‐5p promotes the proliferation, migration, and invasion of ESCC cells.

### 3.6 circ‐TNRC6B suppresses the proliferation, migration, and invasion of ESCC cells via miR‐452‐5p

**Fig. 6:**
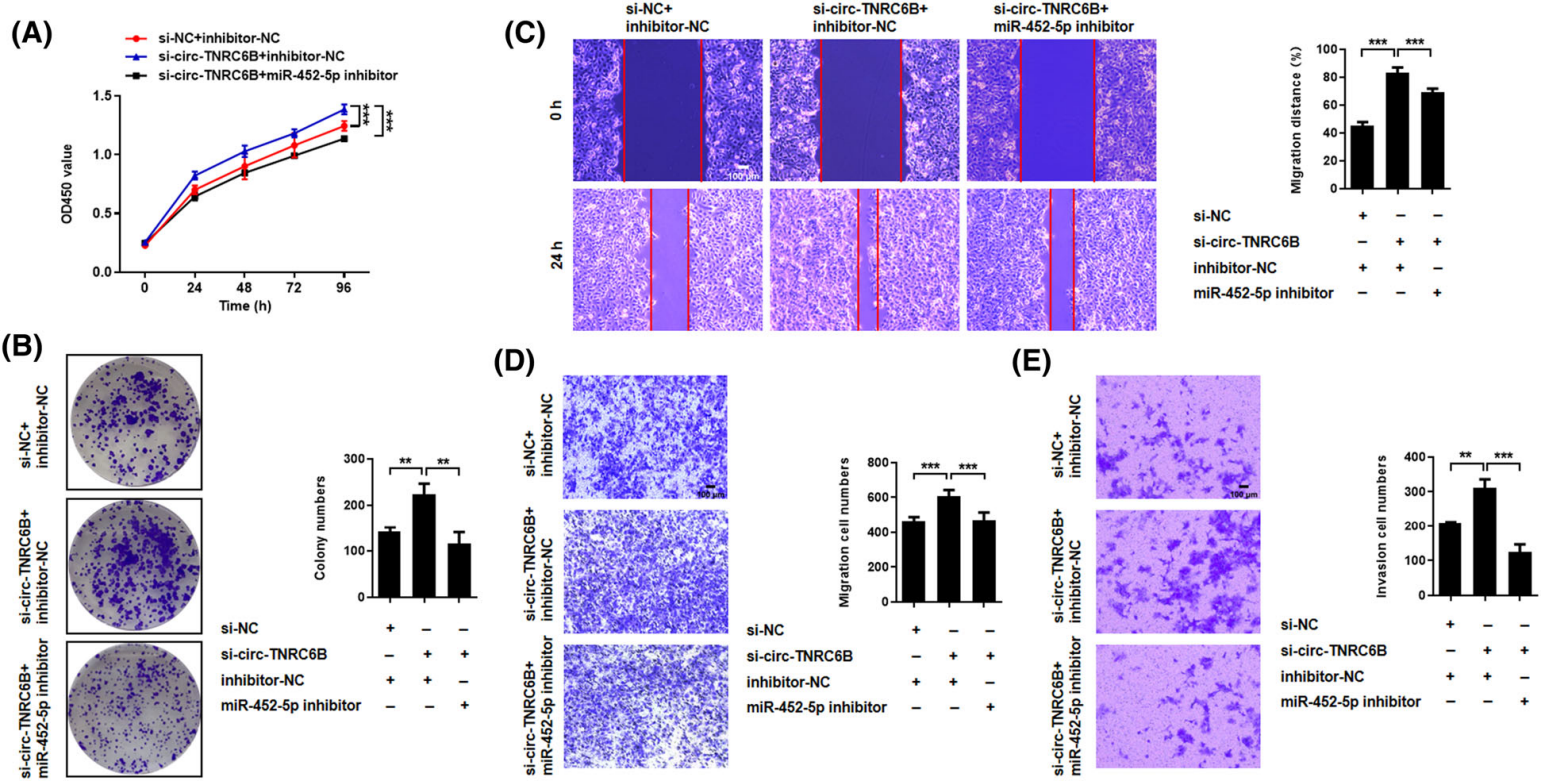
circ‐TNRC6B suppresses proliferation, migration, and invasion abilities of ESCC cells via miR‐452‐5p. (A, B) KYSE150 cells were transfected with negative control plasmid, circ‐TNRC6B siRNA alone, or co‐transfected with circ‐TNRC6B siRNA and miR‐452‐5p inhibitor. CCK‐8 (*n* = 4, *P*‐values were determined by two‐way ANOVA) and colony formation assay (*n* = 3, *P*‐values were determined by one‐way ANOVA) showed silencing circ‐TNRC6B alone significantly promoted the proliferation and clonogenic capacity of KYSE150 cells, and the above promoting effect of circ‐TNRC6B siRNA could be partially reversed by miR‐452‐5p inhibitor. (C–E) Wound‐healing and transwell assay indicated silencing circ‐TNRC6B alone remarkably increased the migration and invasion abilities of KYSE150 cells, while simultaneous knockdown of miR‐452‐5p could partially restore such promoting effects. Data are represented as mean ± SD (*n* = 3, *P*‐values were determined by one‐way ANOVA). Scale bar: 100 μm. **P < 0.01, ***P < 0.001.

Next, the hypothesis that circ‐TNRC6B serves as a miR‐452‐5p sponge and that circ‐TNRC6B exerts its inhibitory biological function through miR‐452‐5p was verified. KYSE150 cells were transfected with NC plasmid or circ‐TNRC6B siRNA or co‐transfected with circ‐TNRC6B siRNA and miR‐452‐5p inhibitor to perform functional rescue experiments. CCK‐8 and colony formation assay demonstrated that circ‐TNRC6B knockdown significantly promoted the proliferative and clonogenic ability of KYSE150 cells. Transfection with miR‐452‐5p inhibitor partially mitigated the circ‐TNRC6B knockdown‐mediated upregulation of cell proliferation and colony formation (Fig. 6A,B). Wound‐healing assay, transwell migration, and invasion analysis indicated that circ‐TNRC6B knockdown markedly increased the migration and invasion of KYSE150 cells. Additionally, miR‐452‐5p knockdown partially mitigated the circ‐TNRC6B knockdown‐mediated upregulation of migration and invasion of KYSE150 cells (Fig. 6C–E). These results indicate that circ‐TNRC6B suppresses the proliferation, migration, and invasion abilities of ESCC cells via miR‐452‐5p.

### 3.7 circ‐TNRC6B inhibits the proliferation and invasion of ESCC cells by regulating the miR‐452‐5p/DAG1 axis

**Fig. 7:**
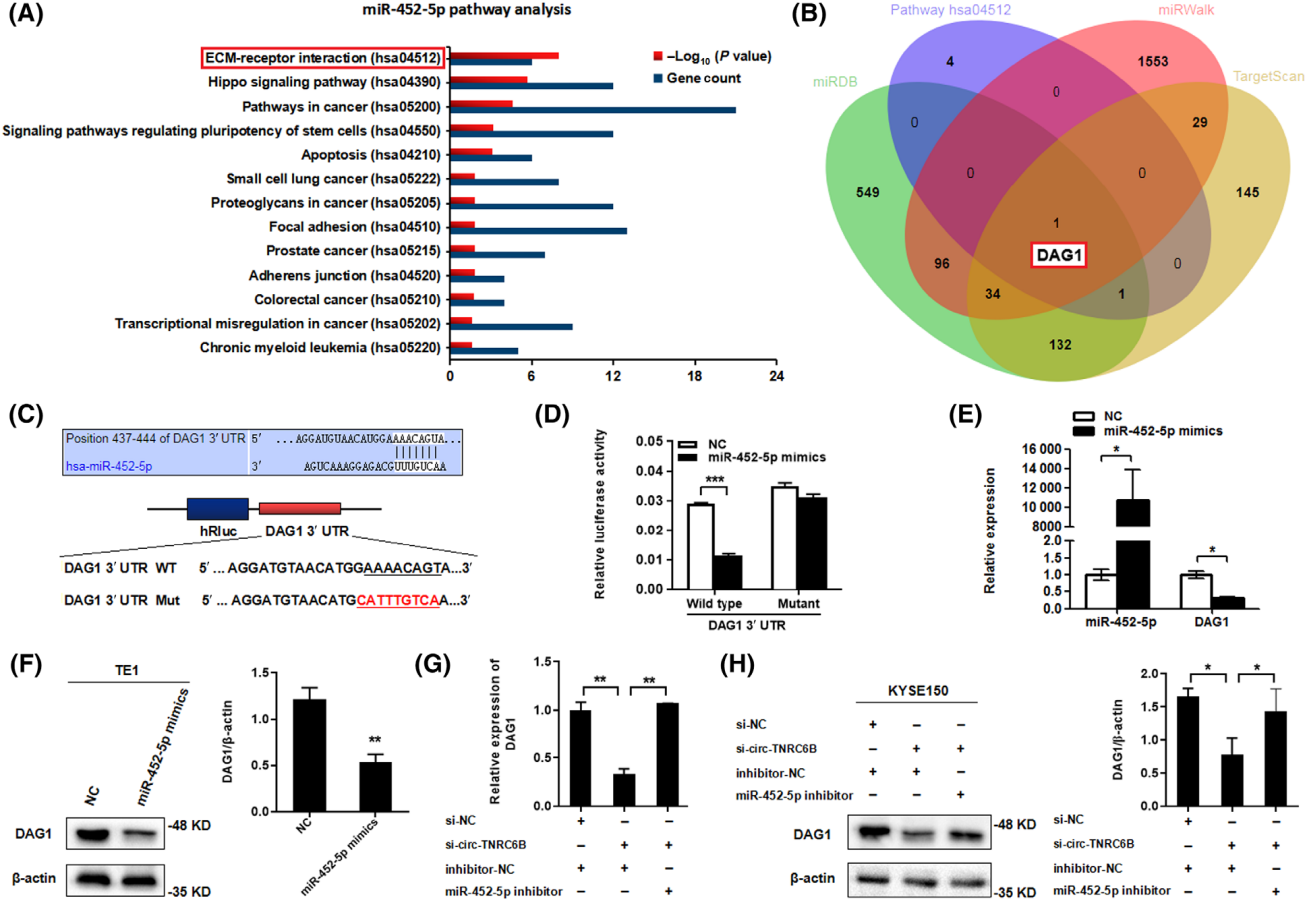
circ‐TNRC6B inhibits the proliferation and invasion of ESCC cells by regulating miR‐452‐5p/DAG1 axis. (A) KEGG pathway analysis of miR‐452‐5p was predicted through TarBase database by DIANA TOOLS. (B) Venn diagram showing the intersection of potential downstream mRNA targets of miR‐452‐5p predicted by Targetscan, miRWalk, miRDB databases, and the enriched gene of ECM‐receptor interaction pathway. (C) The predicted hsa‐miR‐452‐5p binding site in DAG1 3′UTR by Targetscan database, and a schematic representation of the DAG1 3′UTR wild‐type or mutant reporter constructs. (D) Dual‐luciferase reporter assay was performed to detect the luciferase activity of DAG1 3′UTR wild‐type or mutant reporter constructs co‐transfected with miR‐452‐5p mimics or negative control vector. Data are represented as mean ± SD (*n* = 2, *P*‐values were determined by Mann–Whitney *U*‐test). (E, F) DAG1 mRNA and protein levels after miR‐452‐5p overexpression in TE1 cells were detected by qRT‐PCR (*n* = 2, *P*‐values were determined by Mann–Whitney *U*‐test) and western blotting assay (*n* = 3, *P*‐values were determined by Mann–Whitney *U*‐test). Data are represented as mean ± SD. (G, H) DAG1 mRNA and protein levels after silencing circ‐TNRC6B alone or simultaneous knockdown of miR‐452‐5p in KYSE150 cells were detected by qRT‐PCR (*n* = 2, *P*‐values were determined by one‐way ANOVA) and western blotting assay (*n* = 3, *P*‐values were determined by one‐way ANOVA). Data are represented as mean ± SD. *P < 0.05, **P < 0.01, ***P < 0.001.

As circ‐TNRC6B functions as a competing endogenous RNA (ceRNA) for miR‐452‐5p, the underlying molecular mechanism may be related to the downstream target genes of miR‐452‐5p. Data from the TarBase database were retrieved using DIANA TOOLS to predict the downstream Kyoto Encyclopedia of Genes and Genomes (KEGG) pathways of miR‐452‐5p. The results showed that miR‐452‐5p was significantly enriched in several tumor‐related signaling pathways, especially in the ECM‐receptor interaction pathway (Fig. S6; Fig. 7A). The potential downstream target genes of miR‐452‐5p were predicted using the miRWalk, miRDB, and TargetScan databases. The common gene (DAG1) predicted by the three databases and genes enriched in the ECM‐receptor interaction pathway were selected for further investigation (Fig. 7B). DAG1 encodes a cell adhesion molecule that is reported to be involved in the growth, invasion, and aggressive phenotype of various tumors, such as colorectal cancer, prostate cancer, and gastric cancer [27, 28, 29]. In EC, DAG1 expression was significantly downregulated, promoting the progression of the tumor [30]. The binding site of miR‐452‐5p in DAG1 3′UTR was predicted using the TargetScan database. The wild‐type or mutant DAG1 3′‐UTR luciferase reporter vectors were constructed (Fig. 7C). The results of the dual‐luciferase reporter gene assay indicated that miR‐452‐5p mimic transfection significantly decreased the luciferase activity of wild‐type DAG1 3′‐UTR vector when compared to NC transfection but did not affect the luciferase activity of the mutant DAG1 3′‐UTR vector (Fig. 7D). Furthermore, miR‐452‐5p overexpression markedly downregulated the DAG1 mRNA and protein levels (Fig. 7E,F). The effects of circ‐TNRC6B knockdown or circ‐TNRC6B/miR‐452‐5p double knockdown on the mRNA and protein levels of DAG1 were examined in KYSE150 cells using qRT‐PCR and western blotting assay. circ‐TNRC6B knockdown significantly downregulated the DAG1 mRNA and protein levels. Meanwhile, miR‐452‐5p knockdown partially mitigated the circ‐TNRC6B knockdown‐induced downregulation of DAG1 mRNA and protein levels (Fig. 7G,H). Thus, these findings indicate that circ‐TNRC6B inhibits the proliferation and invasion of ESCC cells by regulating the miR‐452‐5p/DAG1 axis.

## 4 Discussion

As early as the 1970s, researchers found that there were pathogenic single‐stranded circular viroids in plants, which was the first time that humans discovered circRNAs [31]. Hsu and Coca‐Prados [32] observed the presence of circRNAs in the cytoplasm of eukaryotic cells under an electron microscope in 1979. Subsequently, *Sry*‐derived and *mbl*‐derived circular transcripts were discovered in mice and Drosophila, respectively [33, 34]. The development of high‐throughput RNA sequencing technology in the last two decades has enabled the identification of several circRNAs. The functions of circRNAs have piqued the interest of the scientific community in the biomedical research field.

Most circRNAs, which are generated by special back‐splicing, are derived from exons of precursor mRNAs and are abundant in the cytoplasm of eukaryotic cells [35]. The expression abundance, stability, and specificity of circRNAs contribute to their roles in a series of biological processes, such as cancer initiation and progression, and as cancer diagnostic and therapeutic biomarkers [36]. This study identified circ‐TNRC6B, which was derived from exons 9–13 of TNRC6B pre‐mRNA and predominantly located in the cytoplasm of ESCC cells. Clinical data analysis of tissue chip array suggested that the expression of circ‐TNRC6B in ESCC tissues was markedly downregulated when compared with that in non‐tumor tissues. Additionally, circ‐TNRC6B downregulation was correlated with advanced T stage and poor prognosis of ESCC patients. These findings suggested that circ‐TNRC6B is related to the occurrence and development of ESCC and can serve as a prognostic biomarker for guiding ESCC treatment.

CircRNAs are reported to function through various mechanisms, including serving as miRNA sponges, protein scaffolds, and transcription factors, interacting with RNA‐binding proteins (RBPs), and encoding peptides or proteins [37]. The miRNA sponging function of circRNAs is the most widely examined molecular mechanism. For example, ciRS‐7 (or CDR1as) and SRY circRNA transcripts function as natural and stable molecular sponges for specific miRNAs [38, 39]. Similarly, circHIPK3 sponges multiple miRNAs, including miR‐124, to regulate the growth of human cells [40]. CircHMGB2 sponges miR‐181a‐5p in lung adenocarcinomas and squamous cell carcinomas to regulate tumor microenvironment and anti‐PD‐1 resistance [41]. Meanwhile, circORC5 was shown to suppress gastric cancer progression by serving as a miR‐30c‐2‐3p sponge to regulate *AKT1S1* expression [42]. Although these circRNAs are expressed in different types of cells, they are preferentially localized to the cytoplasm, which is critical for their miRNA sponging activity. Consistently, circ‐TNRC6B was predominantly localized to the cytoplasm of ESCC cells in this study. Hence, the ability of circ‐TNRC6B to function as a miRNA sponge in ESCC cells was examined. Data from the ENCORI and circBank databases were retrieved to predict miRNAs that potentially bind to circ‐TNRC6B. The common miRNAs predicted by home‐made miRNA target prediction software from Arraystar based on TargetScan and miRanda data were identified. Based on this screening method, miR‐452‐5p was selected as a candidate miRNA that may function as a molecular sponge for circ‐TNRC6B. Furthermore, previously employed experimental techniques [16], such as FISH, RIP, and dual‐luciferase reporter gene analysis were also employed in this study to validate the above hypothesis. circ‐TNRC6B interacted with miR‐452‐5p through a molecular sponging mechanism. One circRNA may interact with multiple miRNAs. The miRNAs explored in this study were screened through bioinformatics prediction, which may be non‐comprehensive and require further investigations.

Recent studies have demonstrated the critical roles of miR‐452‐5p in the pathogenesis of several cancers. In colorectal cancer, miR‐452‐5p promoted tumor progression by targeting *PKN2* and *DUSP6* to activate the ERK/MAPK signaling pathway [43]. miR‐452‐5p facilitated hepatocellular carcinoma cell proliferation, migration, and invasion by targeting *COLEC10* [44]. Additionally, miR‐452‐5p promotes the invasion and metastasis of renal cancer cells by regulating the SMAD4/SMAD7 signaling pathways [45]. Accordant with the findings of these previous studies, miR‐452‐5p promoted the proliferation, migration, and invasion of ESCC cells in this study. Generally, miRNAs exert their effects by directly binding to the 3′‐UTR of downstream target genes, resulting in the degradation or repression of target mRNAs at the post‐transcriptional level [46, 47, 48, 49]. In this study, data from the TarBase database were retrieved using the DIANA TOOLS to analyze KEGG pathways in which miR‐452‐5p are enriched. The common potential target genes of miR‐452‐5p predicted by miRWalk, miRDB, and TargetScan online databases were identified. The common gene DAG1 identified by these databases was selected as a potential target of miR‐452‐5p, which was verified using the dual‐luciferase reporter gene assay. The miR‐452‐5p‐mediated downregulation of DAG1 was confirmed using qRT‐PCR and western blotting assay. miR‐452‐5p may exert oncogenic effects by targeting genes other than DAG1. The downstream target genes of miR‐452‐5p in this study were predicted using computational algorithms and require further investigation.

Rescue experiments were performed to confirm whether circ‐TNRC6B exerts its inhibitory functions through miR‐452‐5p. It was shown that circ‐TNRC6B knockdown markedly increased the proliferation, migration, and invasion of ESCC cells. Meanwhile, miR‐452‐5p knockdown partially reversed the circ‐TNRC6B knockdown‐mediated upregulation of ESCC cell proliferation, migration, and invasion. Additionally, circ‐TNRC6B knockdown significantly downregulated the mRNA and protein levels of DAG1. Moreover, miR‐452‐5p knockdown partially reversed the circ‐TNRC6B knockdown‐mediated downregulation of DAG1 mRNA and protein expression in ESCC cells. DAG1 was reported to function as a tumor suppressor and repress the progression of several cancers, including EC. This study demonstrated the inhibitory effects of circ‐TNRC6B in ESCC cells. Mechanistically, circ‐TNRC6B inhibited the proliferation, migration, and invasion of ESCC cells by regulating the miR‐452‐5p/DAG1 axis.

This study has some limitations. First, the sample size included in the clinical analysis was small, and follow‐up data for several patients were missing. In the future, we will increase the sample size and improve the clinical data collection of patients for detailed analysis. Second, this study transiently knocked down circ‐TNRC6B via siRNA transfection *in vitro*. Experiments with a preclinical animal model with a stable knockout of circ‐TNRC6B *in vivo* were not performed. We attempted to design gRNA in the flanking region of circ‐TNRC6B using clustered regularly interspaced short palindrome repeat (CRISPR)/CRISPR‐associated 9 (Cas9) technology to inhibit circularization. However, the expected effect was not achieved due to the specificity. Third, the mechanism through which circRNAs function as ceRNAs is controversial although some supporting evidence is available [50, 51]. Thus, circ‐TNRC6B may also exert tumor suppressive effects through other mechanisms, such as serving as protein scaffolds and transcription factors, interacting with RBPs, and encoding peptides or proteins, which warrant further investigations.

## 5 Conclusions

This study identified a novel tumor suppressor circRNA called circ‐TNRC6B, which was significantly downregulated in ESCC tissues. circ‐TNRC6B downregulation predicted poor prognosis of patients with ESCC. Functionally, circ‐TNRC6B inhibited the proliferation, migration, and invasion of ESCC cells. Mechanistically, circ‐TNRC6B sponged miR‐452‐5p to upregulate the downstream target gene DAG1 in ESCC cells. The findings of this study suggest that circ‐TNRC6B may serve as a novel prognostic biomarker for ESCC.

## Conflict of interest

The authors declare no conflict of interest.

## Author contributions

LM and MS designed this study. RX, PD, XZ, and ZL performed the experiments. LM, RX, PD, FL, LG, and YZ analyzed and interpreted data. LM drafted this manuscript. RX, LM, and MS corrected this manuscript. All authors have read and approved the final manuscript.

## Supporting information


**Fig. S1.** Negative and positive controls in ESCC cells for FISH assay.
**Fig. S2.** External sequencing data validation of circ‐TNRC6B expression in ESCC.
**Fig. S3.** Correlation analysis of circ‐TNRC6B expression and clinicopathological parameters, and Kaplan–Meier curve for overall survival rate according to different clinicopathological parameters.
**Fig. S4.** Secondary structure of circ‐TNRC6B predicted by RNAfold web server.
**Fig. S5.** miR‐452‐5p promotes the proliferation, migration, and invasion abilities of KYSE150 cells.
**Fig. S6.** KEGG analysis of hsa‐miR‐452‐5p predicted by TarBase database.
**Table S1.** Sequences of primers and probes.
**Table S2.** Association between circ‐TNRC6B expression and the clinicopathological characteristics of ESCC patients.
**Table S3.** Univariate and multivariate Cox regression analysis of factors associated with OS in ESCC.

## Data Availability

All data and materials in this study have been included within the article and Supporting Information.
