# Supplementary material for: Circular RNA circ‐TNRC6B inhibits the proliferation and invasion of esophageal squamous cell carcinoma cells by regulating the miR‐452‐5p/DAG1 axis
Source: Mol Oncol. 2023 Apr 16;17(7):1437–52. doi: 10.1002/1878-0261.13432 (PMC10323880; doi:10.1002/1878-0261.13432)
Supplement: Supplementary file 1 — Fig. S1. Negative and positive controls in ESCC cells for FISH assay. Fig. S2. External sequencing data validation of circ‐TNRC6B expression in ESCC. Fig. S3. Correlation analysis of circ‐TNRC6B expression and clinicopathological parameters, and Kaplan–Meier curve for overall survival rate according to different clinicopathological parameters. Fig. S4. Secondary structure of circ‐TNRC6B predicted by RNAfold web server. Fig. S5. miR‐452‐5p promotes the proliferation, migration, and invasion abilities of KYSE150 cells. Fig. S6. KEGG analysis of hsa‐miR‐452‐5p predicted by TarBase database. Table S1. Sequences of primers and probes. Table S2. Association between circ‐TNRC6B expression and the clinicopathological characteristics of ESCC patients. Table S3. Univariate and multivariate Cox regression analysis of factors associated with OS in ESCC. [file MOL2-17-1437-s001.docx]

**Supplementary Information**

**
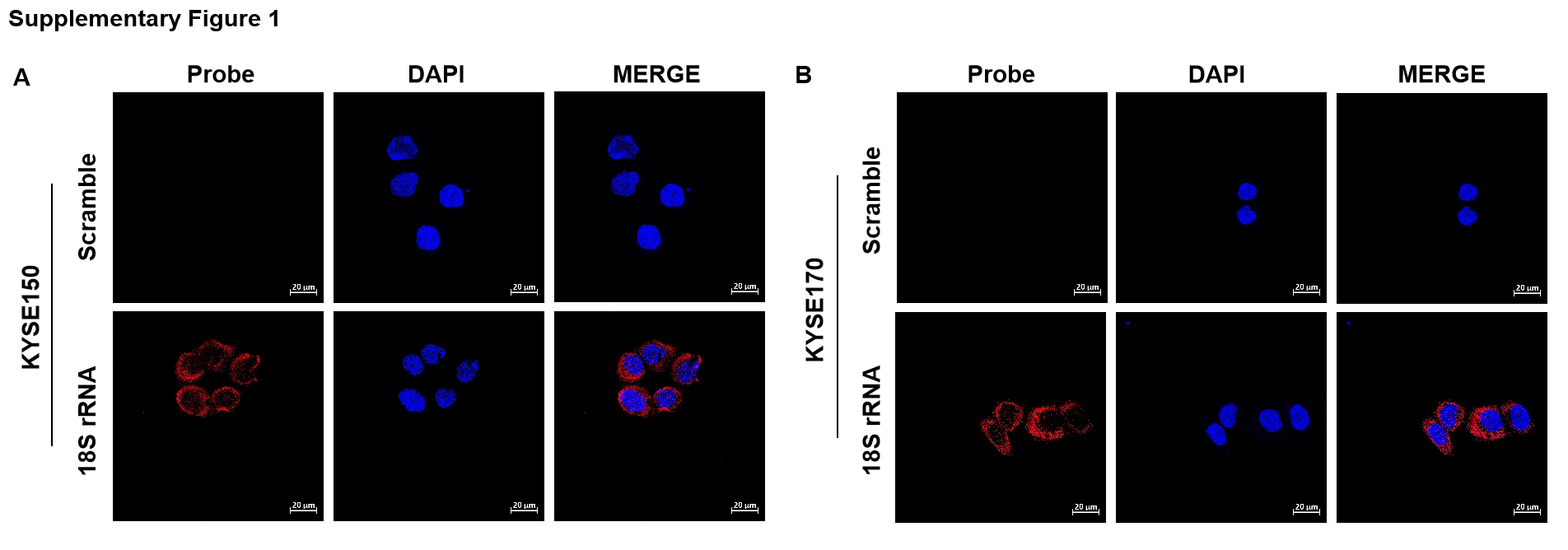
**

**Supplementary Figure 1. The negative and positive controls in ESCC cells for FISH assay.** (A). The representative images of negative and positive controls in KYSE150 cells for FISH assay. (B). The representative images of negative and positive controls in KYSE170 cells for FISH assay. Scramble: negative control; 18S rRNA: positive control.


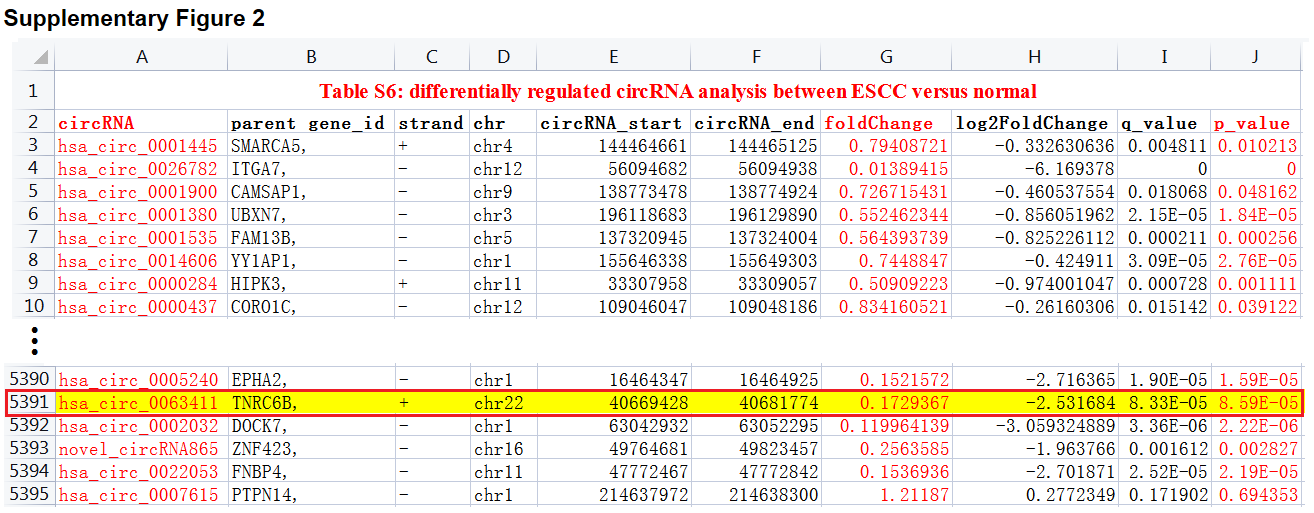


**Supplementary Figure 2. External sequencing data validation of circ-TNRC6B expression in ESCC.** Wang et al. identified differentially regulated circRNAs between ESCC versus normal with RNA sequencing, in which the basic information and differential expression of circ-TNRC6B were highlighted in the red box [23].


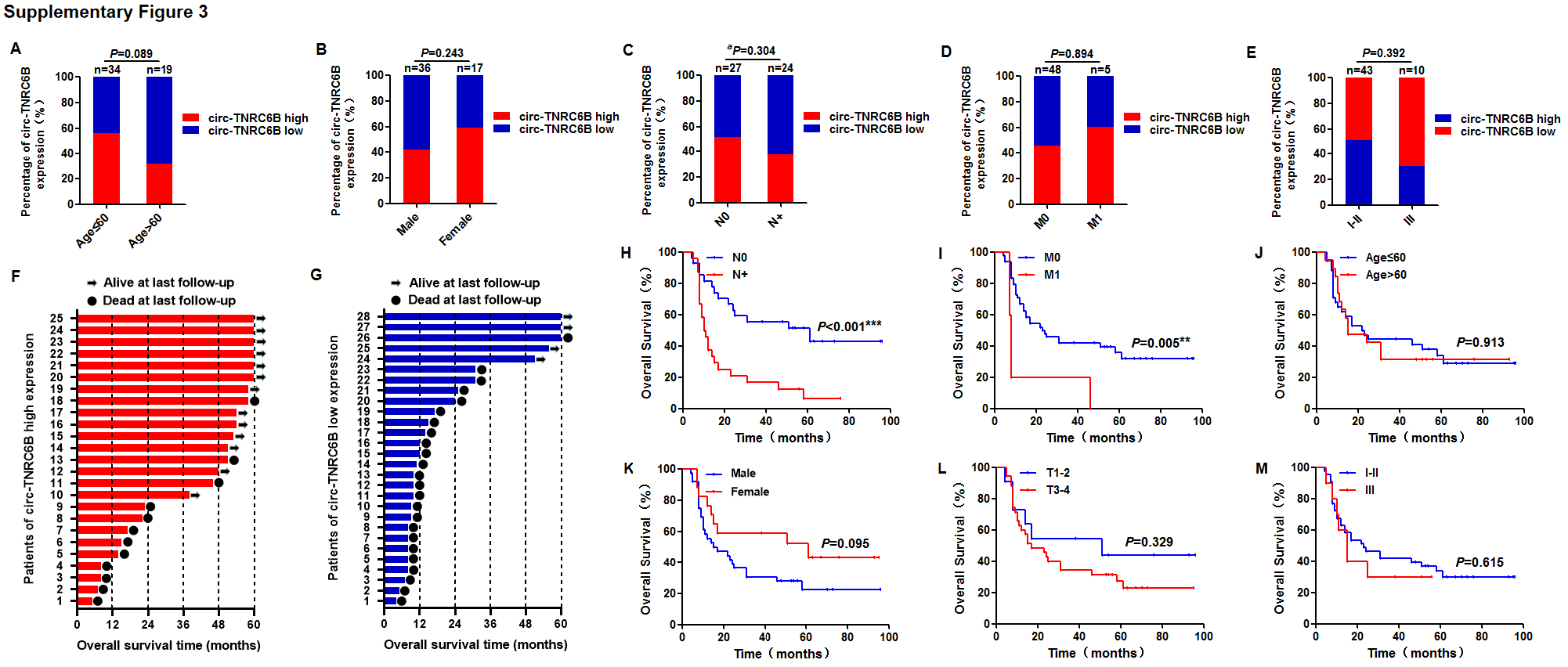


**Supplementary Figure 3. Correlation analysis of circ-TNRC6B expression and clinicopathological parameters, and Kaplan-Meier curve for overall survival rate according to different clinicopathological parameters.** (A-E). The percentage of age, gender, lymph node metastasis, distant metastasis and pathological grade in 25 ESCC tissues with circ-TNRC6B high expression and 28 ESCC tissues with circ-TNRC6B low expression. (F-G). The overall survival time in patients of circ-TNRC6B high and low expression. (H-M). Kaplan-Meier curve for overall survival rate according to lymph node metastasis, distant metastasis, age, gender, T stage, and pathological grade.a Numbers do not equal to the total number due to missing data.


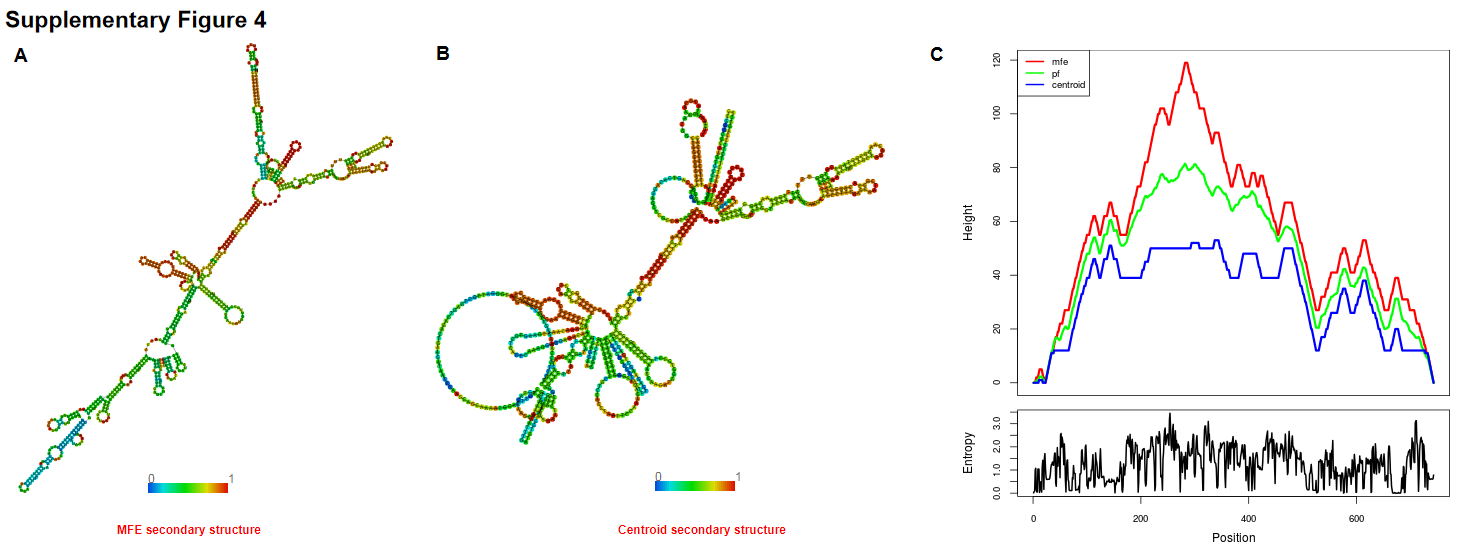


**Supplementary Figure 4. The secondary structure of circ-TNRC6B predicted by RNAfold web server.** (A). The minimum free energy (MFE) of the MFE secondary structure was -238.70 kcal/mol. (B). The MFE of the Centroid secondary structure was -150.00 kcal/mol. (C). The mountain plot representation of the MFE structure, the thermodynamic ensemble of RNA structure, and the centroid structure.


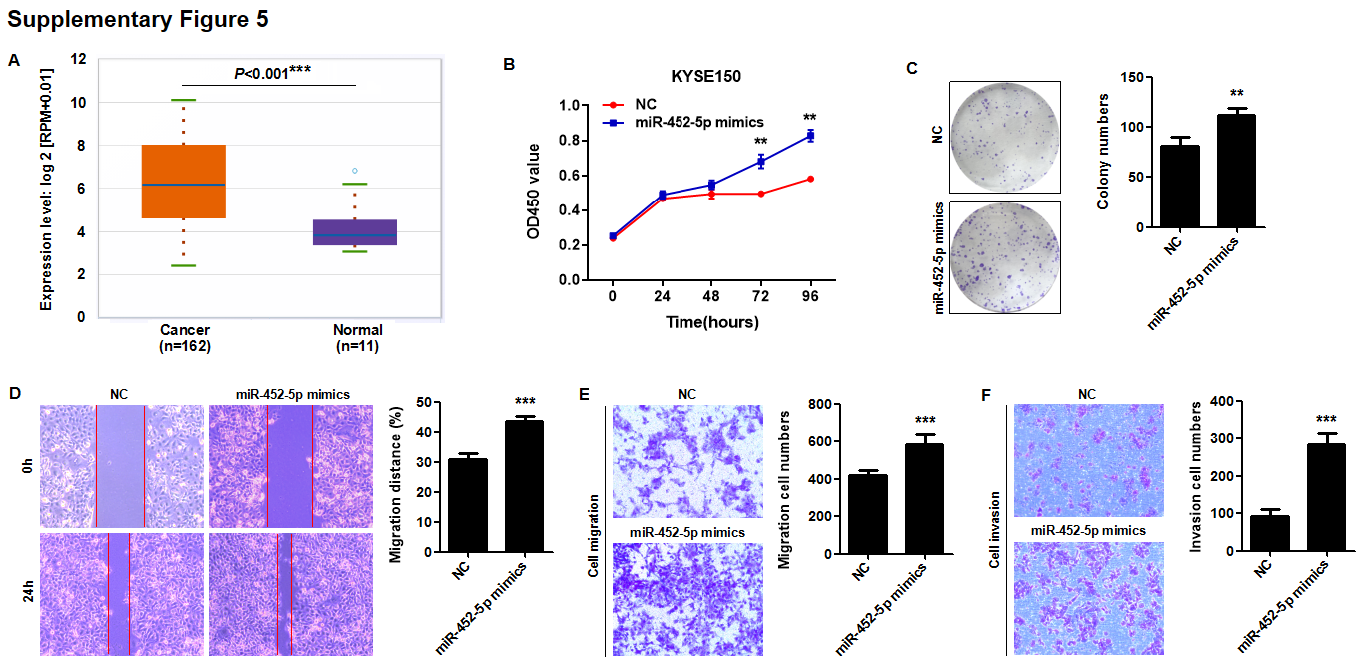


**Supplementary Figure 5.** **miR-452-5p promotes the proliferation, migration, and invasion abilities of KYSE150 cells.** (A). Differential expression of miR-452-5p in esophageal carcinoma tissues and normal esophageal tissues based on ENCORI pan-cancer analysis data integrated from TCGA project. (B-C). The proliferative and clonogenic ability of KYSE150 cells after exogenous miR-452-5p mimics transfection was detected by CCK-8 and colony formation assay. (D-E). The migration ability of KYSE150 cells after exogenous miR-452-5p mimics transfection was detected by wound healing and transwell migration assay. (F). The invasion ability of KYSE150 cells after exogenous miR-452-5p mimics transfection was detected by transwell invasion assay. ***P*<0.01, ****P*<0.001.


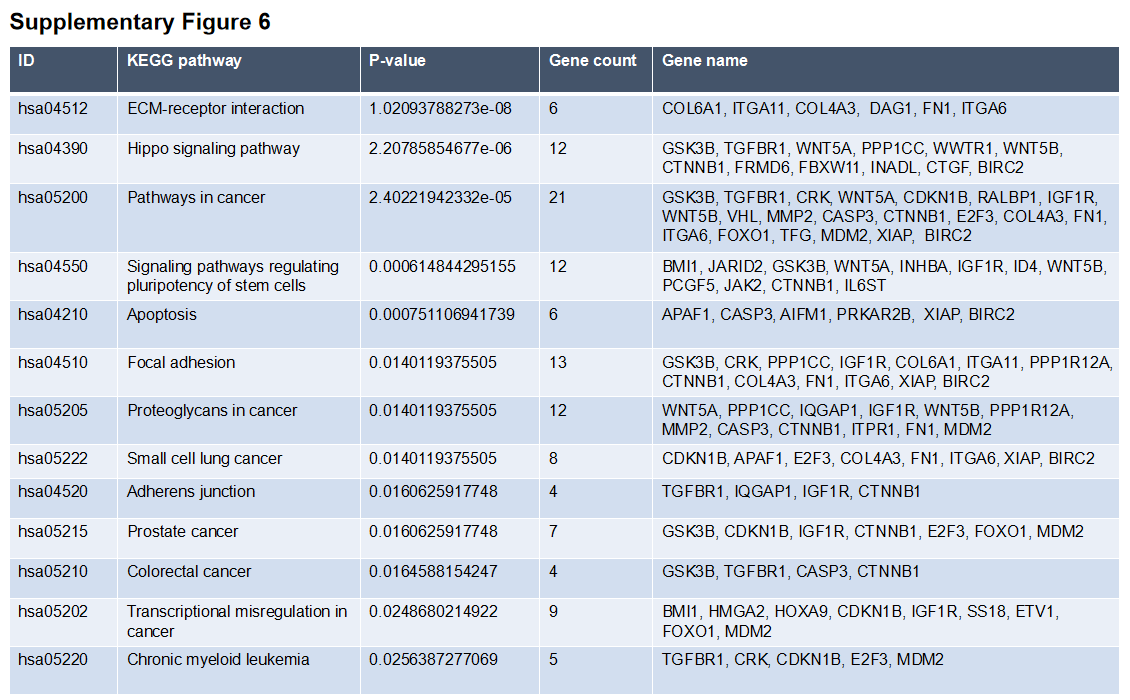


**Supplementary Figure 6. KEGG analysis of hsa-miR-452-5p predicted by TarBase database.** KEGG pathway ID, name, p-value, enriched gene count and gene name of hsa-miR-452-5p predicted through TarBase database by DIANA TOOLS.

**Supplementary Tables**

**Supplementary Table S1 Sequences of primers and probes**

**Primer sequences**

hsa_circ_0063411

Forward primer, 5’-CCGTGCAGCCACTAAATTCT-3’

Reverse primer, 5’-CTCCATCCTCCTCCTCTTCC-3’

TNRC6B-linear mRNA

Forward primer, 5’-TTTGATGTGGACAAGCGAGC-3’

Reverse primer, 5’-TGACGTTGGGTAGTGTGGAA-3’

DAG1

Forward primer, 5’-TTCCTCCCAGACCCTGTCTT -3’

Reverse primer, 5’-ACATCCTCATCCCAGGTCCA-3’

GAPDH

Forward primer, 5’-CGCTGAGTACGTCGTGGAGTC-3’

Reverse primer, 5’-GCTGATGATCTTGAGGCTGTTGTC-3’

**FISH Probe sequences**

hsa_circ_0063411 5’ Biotin-TTTGGAATCTGGGGGGAAATAAACTG-3’ Biotin

hsa-miR-452-5p 5' FAM-TCAGTTTCCTCTGCAAACAGTT-3'

**siRNA** **target** **sequence**

si-hsa_circ_0063411 5’-CCCAGATTCCAAATCTATG-3’

**Supplementary Table S2 The association between circ-TNRC6B expression and the clinicopathological characteristics of ESCC patients [n(%)]**

| Parameters | Groups | N | circ-TNRC6B | | *X*^2^ | *P* |
| --- | --- | --- | --- | --- | --- | --- |
|  |  |  | High (n=25) | Low (n=28) |  |  |
| Age (years) | ≤60 | 34 | 19 (55.9) | 15 (44.1) | 2.889 | 0.089 |
|  | >60 | 19 | 6 (31.6) | 13 (68.4) |  |  |
| Gender | Male | 36 | 15 (41.7) | 21 (58.3) | 1.364 | 0.243 |
|  | Female | 17 | 10 (58.8) | 7 (41.2) |  |  |
| T stage**^a^** | T1-2 | 11 | 9 (81.8) | 2 (18.2) | 6.695 | 0.010* |
|  | T3-4 | 35 | 13 (37.1) | 22 (62.9) |  |  |
| Lymph node metastasis**^a^** | N0 | 27 | 14 (51.9) | 13 (48.1) | 1.057 | 0.304 |
|  | N+ | 24 | 9 (37.5) | 15 (62.5) |  |  |
| Distant metastasis | M0 | 48 | 22 (45.8) | 26 (54.2) | 0.018 | 0.894**^b^** |
|  | M1 | 5 | 3 (60.0) | 2 (40.0) |  |  |
| Pathological grade | I-II | 43 | 22 (51.2) | 21 (48.8) | 0.733 | 0.392**^b^** |
|  | III | 10 | 3 (30.0) | 7 (70.0) |  |  |

^a^Numbers do not equal to the total number due to missing data

^b^Continuity correction

**P*<0.05

**Supplementary Table S3 Univariate and multivariate Cox regression analysis of factors associated with OS in ESCC**

| Variables | Univariate analysis | | Multivariate analysis | |
| --- | --- | --- | --- | --- |
|  | HR (95% CI) | *P* | HR (95% CI) | *P* |
| Age (years) |  | 0.913 |  |  |
| ≤60 | Reference |  |  |  |
| >60 | 1.038 (0.524–2.056) |  |  |  |
| Gender |  | 0.095 |  |  |
| Male | Reference |  |  |  |
| Female | 0.542 (0.276–1.061) |  |  |  |
| T stage |  | 0.329 |  |  |
| T1-2 | Reference |  |  |  |
| T3-4 | 1.533 (0.697–3.373) |  |  |  |
| Lymph node metastasis |  | 0.000*** |  | 0.005** |
| N0 | Reference |  | Reference |  |
| N+ | 2.992 (1.505–5.951) |  | 2.738 (1.346–5.570) |  |
| Distant metastasis |  | 0.005** |  | 0.017* |
| M0 | Reference |  | Reference |  |
| M1 | 3.416 (0.708–16.490) |  | 3.659 (1.257–10.646) |  |
| Pathological grade |  | 0.615 |  |  |
| I-II | Reference |  |  |  |
| III | 1.227 (0.508–2.964) |  |  |  |
| circ-TNRC6B expression |  | 0.002** |  | 0.004** |
| Low | Reference |  | Reference |  |
| High | 0.360 (0.186–0.698) |  | 0.321 (0.149–0.691) |  |

Abbreviations: CI, confidence interval; HR, hazard ratio.

**P*<0.05，***P*<0.01，****P*<0.001
